# Innovative approaches to English pronunciation instruction in ESL contexts: integration of multi-sensor detection and advanced algorithmic feedback

**DOI:** 10.3389/fpsyg.2024.1484630

**Published:** 2025-01-22

**Authors:** Li Ping, Ning Tao

**Affiliations:** ^1^School of Foreign Languages, Jiangsu Ocean University, Lianyungang, China; ^2^School of Computer Engineering, Guilin University of Electronic Technology, Beihai, China

**Keywords:** accuracy, English as a second language, English pronunciation, feedback neural network, speech signal processing, teaching evaluation

## Abstract

**Introduction:**

Teaching English pronunciation in an English as a Second Language (ESL) context involves tailored strategies to help learners accurately produce sounds, intonation, and rhythm.

**Methods:**

This study presents an innovative method utilizing advanced technology and algorithms to enhance pronunciation accuracy, fluency, and completeness. The approach employs multi-sensor detection methods for precise data collection, preprocessing techniques such as pre-emphasis, normalization, framing, windowing, and endpoint detection to ensure high-quality speech signals. Feature extraction focuses on key attributes of pronunciation, which are then fused through a feedback neural network for comprehensive evaluation. The experiment involved 100 college students, including 50 male and 50 female students, to test their English pronunciation.

**Results:**

Empirical results demonstrate significant improvements over existing methods. The proposed method achieved a teaching evaluation accuracy of 99.3%, compared to 68.9% and 77.8% for other referenced methods. Additionally, students showed higher levels of fluency, with most achieving a level of 4 or above, whereas traditional methods resulted in lower fluency levels. Spectral feature analysis indicated that the amplitude of speech signals obtained using the proposed method closely matched the original signals, unlike the discrepancies found in previous methods.

**Discussion:**

These findings highlight the effectiveness of the proposed method, showcasing its ability to improve pronunciation accuracy and fluency. The integration of multi-sensor detection and neural network evaluation provides precise results, outperforming traditional approaches.

## 1 Introduction

English pronunciation instruction has consistently been a significant focus in the ESL setting. This emphasis is driven by the understanding that accurate pronunciation is crucial for effective communication and integration into English-speaking contexts (Azimova and Solidjonov, 2023). Conventional methods of teaching English pronunciation typically prioritize phonetic symbols and pronunciation norms. These traditional approaches often involve the use of phonetic charts (Alghazo et al., 2023), repetitive drills, and the memorization of pronunciation rules, which aim to familiarize learners with the standard sounds of the English language. Because learners’ mother tongue background, language context, learning habits and motivation are different, traditional pronunciation teaching methods are often difficult to meet the needs of all learners (Clymer et al., 2020). The theory of second language acquisition provides us with important theoretical support. This theory holds that language acquisition is a complex process involving multiple factors such as the learner’s age, cognitive ability, learning motivation, and the quality and quantity of language input. Among them, the quality and quantity of language input have a significant impact on the accuracy of pronunciation (Alghazo et al., 2019). Extensive exposure and use of the target language, especially through interaction with native speakers, can help learners better perceive and imitate the pronunciation characteristics of English. However, in ESL contexts, learners often lack sufficient language input and practical opportunities. They do not provide tailored assistance for individual variations and accent replication (Yaccob et al., 2023). Every learner comes with a unique set of phonetic challenges influenced by their native language, individual speech habits, and personal learning pace. For instance, a Spanish speaker might struggle with English vowel sounds, while a Chinese speaker might find it difficult to pronounce consonant clusters. Traditional methods often fail to address these specific issues, leading to persistent pronunciation errors and frustration among learners. Furthermore, conventional instruction does not adequately account for accent replication. Learners might be able to produce isolated sounds correctly but still struggle with the fluid, natural intonation patterns, stress, and rhythm that characterize native speech (Adelekea and Onyebuchib, 2023). This gap in teaching can result in speech that, while technically correct, still sounds unnatural or stilted to native speakers. Therefore, there is a pressing need for innovative instructional methods that go beyond the one-size-fits-all approach. These methods should incorporate personalized guidance, allowing educators to address the specific phonetic challenges and learning styles of individual students. Moreover, they should facilitate accent replication by integrating practices that focus on the suprasegmental aspects of pronunciation, such as intonation, stress, and rhythm, ensuring that learners can achieve a more natural and fluent English speech (Gao et al., 2023). Many learners face difficulties with English pronunciation due to differences in their native language contexts and congenital phonetic conditions. Traditional teaching methods often do not address these unique challenges. Therefore, exploring innovative approaches, such as advanced technology and targeted pronunciation techniques, is crucial for developing effective, personalized instruction and improving learners’ pronunciation skills (Priya and Kumar, 2020). The innovative teaching method that combines modern technology, related algorithms, personalized guidance, and real-time feedback offers a promising solution for overcoming pronunciation barriers. By using tools like speech recognition systems and interactive phonetic training software, educators can provide immediate, detailed feedback, allowing learners to quickly correct errors and improve their pronunciation. This approach tailor’s instruction to individual phonetic challenges and linguistic backgrounds, enhancing pronunciation accuracy and boosting confidence. As learners master accurate sound production, intonation, and stress patterns, they can integrate more effectively into English-speaking contexts, leading to improved fluency and overall communication effectiveness. Currently, English pronunciation teaching methods primarily include recording and playback, visual aids, and electronic learning tools. However, these approaches have notable limitations. While recording and playback enable students to compare their pronunciation with models, they lack the immediacy of real-time feedback and corrective guidance. Visual aids can illustrate oral morphology, but non-native speakers often struggle with imitation despite these visual representations. The advancement of electronic learning tools has significantly enhanced personalized pronunciation instruction; however, the accuracy of some systems still requires refinement. This indicates that current English pronunciation teaching methods have their limitations (Liu, 2021). In order to improve students’ pronunciation accuracy and fluency, these methods should incorporate advanced technological solutions to offer personalized guidance and real-time feedback, thereby addressing the specific needs of learners and improving their English pronunciation skills in the ESL context.

### 1.1 Literature review

Wen (2020) introduced a method for automatically correcting English pronunciation errors. The system is based on the DTW algorithm and involves optimizing voice recognition sensors and improving speech recognition processors to construct the hardware. Develop and extract parameters that indicate errors in English pronunciation using the English pronunciation collecting tool, and finalize the software design of the system. An automatic correction solution for English pronunciation issues has been developed using the DTW algorithm. However, this study lacks evaluation and analysis of English pronunciation quality, which hinders the effective improvement of pronunciation teaching. Tuba et al. (2018) explored data mining and clustering, focusing on web intelligence, a field facing challenges due to complex, dynamic, unstructured, and large data. Traditional clustering methods were insufficient, prompting the authors to propose a novel approach combining the bare bones fireworks algorithm and k-means clustering. This hybrid method aimed to improve web intelligence data clustering. Li (2020) analyzed the drawbacks of traditional multimedia teaching systems and highlighted the benefits of network-based systems. He focused on designing an intelligent central control multimedia teaching system and discussed using advanced technologies like remote control and streaming media in teaching practices, demonstrating their potential to transform educational contexts efficiently and effectively. Niu and Wei (2023) aimed to improve speech denoising accuracy and robustness by proposing an adaptive noise reduction method based on noise classification. They created a new acoustic feature matrix combining LogFbank features and perceptual linear prediction coefficients and designed a noise classifier using a support vector machine. To address “music noise” issues in traditional methods, they adaptively updated the voice activity detection threshold based on background noise types and optimized parameters for a noise estimation algorithm. Li and Wu (2023) explored the impact of emerging technologies like cloud computing, mobile computing, and AI on higher education, emphasizing the need for digital transformation. As China’s higher education system reached popularization, they highlighted the importance of digitalization to meet the demands for diverse quality, lifelong learning, personalized training, and modern governance. To address these needs, Li and Wu developed an embedded voice teaching system using cloud computing and a deep learning model. This system aimed to improve existing university teaching methods and enable ubiquitous learning. They integrated Hidden Markov Models (HMM) and Long Short-Term Memory (LSTM) networks to enhance voice recognition performance, improving recognition rates, anti-interference, and noise robustness. Experimental tests showed high recognition accuracy and noise immunity, confirming the system’s stability and reliability. Feedback from trials indicated that the new system significantly enhanced the intelligence and adaptability of college teaching methods, promoting improvements in students’ cultural literacy and innovation abilities. Jiang and Chen (2024) explored the practical implications of machine-assisted translation, addressing challenges in parameter acquisition and model architecture. Their study used Python data analysis to simulate a translation model, introducing a novel approach that integrated the source syntax tree into the encoder-decoder paradigm. They designed an encoder with a bidirectional GRU RNN, processing syntax tree information from the root node downward. Simulation results showed that for sentences longer than 20 words, their model significantly improved performance, especially for sentences over 25 words. The retrained model quickly restored baseline accuracy with minimal precision loss, demonstrating effective model compression. Bardol (2023) presented a reflective analysis of a pedagogical innovation in applied linguistics through an action research project aimed at improving English pronunciation teaching in a blended learning system at the University of the Antilles in Martinique. Conducted over 3 years (2017–2020), this experiment targeted language students in professional disciplines. The chapter detailed the contextual conditions, key actors, implementation strategies, and obstacles encountered. Bardol also provided reflection tools and recommendations for researchers looking to adapt this innovation to other contexts. Nguyen (2024) explored the rising interest in pronunciation research in English language teaching, focusing on textbook content, teachers’ beliefs, and learners’ attitudes. Despite evidence supporting communicative pronunciation teaching for improving L2 learners’ intelligibility, recent studies showed it remained unsystematic, often relying on recasts and prompts. This inconsistency likely stemmed from a lack of guidance for teachers. Nguyen’s chapter critically reflected on an innovation in Vietnamese tertiary EFL classrooms, shifting from error correction to a communicative approach. The chapter detailed the motivation, context, and implementation by six Vietnamese EFL teachers, discussing successes and challenges. It concluded with discussion questions to link with other chapters and understand the innovation drivers. Al-Asi (2024) examined the difficulties and approaches involved in instructing non-native speakers in English pronunciation. This included addressing concerns such as disparities in speech sounds, intonation, patterns of emphasis, and individual variations. The study presented efficacious techniques, including clear instruction, visual and aural assistance, and communicative exercises. Al-Asi highlighted the significance of error correction and feedback in assisting learners in recognizing and rectifying pronunciation faults. The research also addressed the incorporation of pronunciation instruction into a wider English teaching framework, so improving learners’ understanding, precision, fluency, and self-assurance. In summary, Al-Asi emphasized the significance of teaching pronunciation in enhancing one’s ability to communicate effectively, as well as developing listening and speaking abilities, and overall fluency in the English language. Ozodova (2024) explored the methodologies of teaching pronunciation in ESL, focusing on the challenges learners faced, particularly the transfer of phonetic features from their native languages. She highlighted that pronunciation included both segmental (individual sounds) and suprasegmental (stress, rhythm, intonation) features. Historically, instruction focused on individual sounds, but with the rise of communicative language teaching, the emphasis shifted to suprasegmental elements. Contemporary teaching now recognizes the importance of both for achieving intelligibility. Озодова presented exercises like reverse dictation and sound-focused activities, and analyzed common errors such as the substitution of interdental consonants and mispronunciation of silent letters. She underscored the need for a balanced approach to teaching pronunciation to improve students’ overall communicative competence in English. Thi-Nhu Ngo et al. (2024) examined the effectiveness of automatic speech recognition (ASR) on ESL/EFL student pronunciation, analyzing data from 15 studies conducted between 2008 and 2021. They found that ASR had a medium overall effect size (*g* = 0.69). Key findings included that ASR with explicit corrective feedback was highly effective, while indirect feedback showed moderate effectiveness. ASR significantly improved segmental pronunciation but had minimal impact on suprasegmental features. Medium to long treatment durations with ASR yielded better outcomes, whereas short durations were no more effective than non-ASR conditions. Practicing with peers in an ASR setting produced substantial benefits, while practicing alone had smaller effects. ASR was particularly effective for adult learners (18+) and those at an intermediate proficiency level. Ngo et al. concluded that ASR was a valuable tool for L2 pronunciation development and recommended its integration into language learning programs. Yang (2023) underscored the importance of phonetics in language acquisition, asserting that improved pronunciation enhances listening and speaking skills. Despite this, non-English majors often struggled with pronunciation and motivation due to environmental constraints and traditional teaching methods. Yang emphasized the need for innovative phonetic instruction, presenting strategies based on literature reviews and personal experience. These included using real-life scenarios and technology, promoting cooperative learning, and gamifying phonetic instruction. Yang argued that these methods could significantly improve phonetic sensitivity, pronunciation accuracy, listening skills, and oral fluency, thereby creating a more effective learning context for non-English majors.

### 1.2 Novelty and contributions

The novelty and contributions of this work lie in the development of an innovative method for teaching English pronunciation in an ESL context. The study introduces a comprehensive approach that integrates modern technology and advanced algorithms to provide personalized guidance and real-time feedback. Key contributions include the use of multi-sensor detection methods for accurate data collection and scale decomposition of speech signals, advanced signal processing techniques such as pre-emphasis, normalization, framing, windowing, and endpoint detection to optimize speech signal quality, and the extraction and fusion of features related to accuracy, fluency, and completeness using a feedback neural network. Additionally, the development of a computer-assisted evaluation system that leverages multi-feature fusion results to provide detailed feedback enhances the effectiveness of pronunciation teaching. Empirical validation through experimental results demonstrates that the proposed method surpasses existing techniques in terms of accuracy and application effectiveness, significantly improving students’ pronunciation fluency and overall teaching quality. These innovations provide a scientific basis for optimizing and personalizing English pronunciation teaching methods, making them more effective and adaptable to individual learner needs.

The continuation of the article is as follows:

Section two: Optimization of English Pronunciation Teaching Methods, Section Three: Experimental verification analysis, and Section four: Conclusion.

## 2 Optimization of English pronunciation teaching methods

In the field of English pronunciation teaching methods (Alghazo, 2015), advanced research techniques have significantly improved the ability to assess and enhance learners’ pronunciation skills. One critical advancement is the detection of English pronunciation speech signals, which allows for the precise capture of waveform data from learners’ spoken output. This technology enables real-time monitoring and recording of pronunciation behaviors, providing valuable insights into how students produce and articulate sounds (Ruan, 2023). Speech signal preprocessing plays a crucial role in this process, as it ensures the accuracy and stability of the data for subsequent analysis. By cleaning and normalizing the raw speech signals, preprocessing eliminates noise and irregularities, which helps maintain the integrity of the data and supports more reliable analyses. The next step involves feature extraction, where key acoustic properties of the speech signals are analyzed. This stage focuses on identifying and extracting essential features such as pitch, volume, and spectral characteristics (Li and Huang, 2024). These features are critical for understanding the nuances of pronunciation and provide the necessary data for in-depth analysis and evaluation. The extracted speech features are then evaluated to assess various aspects of pronunciation, including accuracy, fluency, and overall quality. This evaluation helps determine the effectiveness of pronunciation teaching methods and offers a detailed assessment of students’ pronunciation levels. By analyzing these features, educators can identify specific areas where students may need additional support or improvement (Nagle and Hiver, 2023). Ultimately, this comprehensive process converts complex speech signals into quantifiable feature information, offering a scientific basis for optimizing and personalizing English pronunciation instruction. This data-driven approach supports the development of tailored teaching strategies, ensuring that instruction meets the individual needs of learners and enhances the overall effectiveness of pronunciation teaching methods (Li, 2024).

### 2.1 English pronunciation speech signal detection

Accurate detection of learners’ pronunciation, identification of specific errors, and targeted feedback are crucial for improving English-speaking proficiency. This process starts with constructing a model to detect and analyze English pronunciation signals using advanced technology. Multi-sensor techniques are then used to collect the raw data (Duan and He, 2023a). These techniques employ sensors to capture detailed aspects of spoken English, such as pitch, volume, and articulation. The collected data undergoes scale decomposition to analyze different frequency ranges and temporal aspects of pronunciation. This process isolates specific features of the speech signal, revealing pronunciation patterns and errors. Detailed analysis then identifies and quantifies pronunciation errors, including incorrect articulation, stress patterns, and intonation issues (Lounis et al., 2024). The results offer valuable insights into the accuracy of learners’ pronunciation and highlight areas that require improvement. By integrating these steps—model construction, multi-sensor data collection, scale decomposition, and detailed analysis—educators can obtain comprehensive and precise feedback on learners’ pronunciation. This feedback is crucial for correcting errors and guiding learners toward more accurate and effective pronunciation. Ultimately, this approach enhances learners’ English-speaking skills by providing targeted interventions that address their specific pronunciation challenges (Duan and He, 2023).

The mathematical model expression for the pronunciation and speech signal of spoken English is given in Equation (1) (Weng et al., 2021):


(1) $$h\left(t\right)=d\left(t\right)+f\left(t\right)⊗u\left(t\right)$$

Among them, $d\left(t\right)$ represents the amplitude of the received signal; $f\left(t\right)$ represents the step transfer function of speech signals; $u\left(t\right)$ represents the resonance peak.

Using this model for English spoken pronunciation speech signal detection and recognition, the distribution of the sampled elements of the speech information is $z_{n}$ , and the echo pulse of the English spoken pronunciation speech signal is represented as Equation (2) (Ma et al., 2021):


(2) $$k\left(t\right)=\int s\left(\phi ,\varepsilon \right)\exp\left[\pi \phi t\right]\times z_{n}y\left(t-\phi \right)dt$$

Among them, $s\left(\phi ,\varepsilon \right)$ represents the output extension function of English spoken pronunciation speech signals; $y\left(t-\phi \right)$ represents the complex envelope of the frequency components of the English spoken pronunciation speech signal; ε represents the bandwidth of signal acquisition feature expansion; ϕ represents the frequency shift feature of English spoken pronunciation speech signals.

When receiving a linear frequency modulation signal, the result of separating English spoken pronunciation speech features is as Equation (3) (Ma et al., 2021):


(3) $$k^{\prime }\left(t\right)=\int \mu \left(a,b\right)\times y\left(t\right)k\left(t\right)\times \frac{1}{\sqrt{\left|a\right|}}dt$$

Among them, $y\left(t\right)$ represents the instantaneous frequency estimation value of the received English spoken pronunciation speech signal; $\mu \left(a,b\right)$ represents the delay component of broadband signal incident on the array element; a represents the high-order statistical feature information of the signal; b represents the frequency shift distribution. At the new cluster head node, the feature components of English spoken pronunciation information obtained are as Equation (4) (Ma et al., 2021):


(4) $$Y_{k}\left(t\right)=\overset{+\infty }{\underset{-\infty }{\int }}R_{k}\left(t\right)k^{\prime }\left(t\right)dt$$

Among them, $R_{k}\left(t\right)$ represents the phase of speech detection.

Update the fusion weights to obtain the output signal component $Y_{k}^{\prime }\left(t\right)$ , which is represented as Equation (5) (Ma et al., 2021):


(5) $$Y_{k}^{\prime }\left(t\right)=\sqrt{\frac{1-h\left(t\right)+Y_{k}\left(t\right)}{2\pi }}$$

Thus, statistical information modeling of English spoken pronunciation speech signals is achieved, and the detection results of English pronunciation speech signals are obtained, providing a foundation for speech signal processing and feature extraction.

### 2.2 English pronunciation speech signal preprocessing

Preprocessing is a crucial initial step and foundational element in speech comparison and recognition, playing a vital role in the effective extraction of speech signal features. During the preprocessing stage of English pronunciation speech signal detection, as detailed in Section 2.1, it is essential to extract feature parameters that accurately capture the core aspects of English pronunciation. This preprocessing phase encompasses several key processes, including pre-emphasis to enhance signal clarity, normalization to standardize the amplitude, framing to segment the speech signal into manageable pieces, windowing to reduce signal distortion, and endpoint detection to identify the precise start and end points of speech segments. Together, these processes ensure that the speech signal is optimally prepared for accurate and meaningful analysis.

#### 2.2.1 Pre emphasis and normalization of speech signals

The purpose of pre-emphasis is to amplify high-frequency components of a speech signal and achieve a flatter frequency spectrum. This is necessary because the power spectrum of speech signals decreases with increasing frequency, leading to most of the energy being concentrated in the lower frequency range. As a result, high-frequency components often have a much lower signal-to-noise ratio. Various external factors, including the effects of the mouth, nose, lips, and airflow on the vocal cords, can further attenuate high-frequency signals during speech production. Pre-emphasis addresses this issue by boosting these high-frequency components to improve their clarity. This process can be implemented using either analog or digital methods. The method of pre-emphasis using a first-order digital filter is implemented, and the formula is as Equation (6) (Le-Qing, 2011):


(6) $$X\left(z\right)=1-\alpha z^{-1}$$

Among them, α represents the pre-emphasis coefficient. If $x\left[n\right]$ is a speech signal, the formula for pre-emphasis is presented in Equation (7) (Yegnanarayana and Gangashetty, 2011):


(7) $$y\left[n\right]=x\left[n\right]-\alpha x\left[n-1\right]$$

The purpose of normalization is to avoid negative effects on speech signal processing caused by the strength of the tester’s pronunciation or the distance between the tester and the recording device. It fixes speech signals with different amplitudes within a similar range for subsequent processing of speech signals. The normalization formula is presented using Equation (8) (Yegnanarayana and Gangashetty, 2011):


(8) $$x\left[n\right]=x\left[n\right]/\max\left(x\left[1,2,3,..,N\right]\right)$$

Among them, $x\left[n\right]$ represents the signal of the n -th sample point of the speech sample; max represents the maximum value of speech sample $x\left[n\right]$ .

#### 2.2.2 Framing and windowing processing of speech signals

Speech signals are typically continuous and vary over time, making them effectively infinite in length. However, over brief intervals—such as 0 ms to 25 ms—the variations in the speech signal are minimal, allowing these short periods to be approximated as steady-state signals. To analyze the speech signal effectively, it is essential to divide it into frames, which involves segmenting the signal into equal time intervals. This framing ensures that each segment is treated consistently along the timeline. To maintain smooth transitions and continuity between adjacent frames, an overlap is introduced, known as the frame shift. The duration of data within each frame is referred to as the frame length, and the frame shift is typically set to a ratio between 0 and 0.5 of the frame length. The frame rate $f\left(n\right)$ can be calculated by the Equation (9) (Quatieri, 2002):


(9) $$f\left(n\right)=\frac{\left|n-s\right|}{\left|l-s\right|}$$

Among them, l represents the frame length; s represents frame shift; n represents the number of voice points.

For the framing operation of speech signals, a moving window function is generally required. Assuming that the speech signal is $X\left(n\right)$ , this value is infinitely long in practical applications. Therefore, it is necessary to perform framing operation on it. The formula is as Equation (10) (Yegnanarayana and Gangashetty, 2011):


(10) $$X_{c}\left(n\right)=X\left(n\right)\times c\left(n-m\right)$$

Among them, $X\left(n\right)$ represents the speech signal; $c\left(n-m\right)$ represents the window function. Generally speaking, choosing different window functions will have different effects on the analysis of speech signals. For example, choosing a wider window function makes the signal smoother, and the window function generally has low-pass characteristics. Choosing different window functions for speech signals will result in different broadband and spectrum leaks. For window functions, rectangular windows are prone to losing the high-frequency part of the signal and the details of the speech signal. However, the advantage is that they have good smoothness, and rectangular windows are generally suitable for time-domain analysis. The smoothness of the Hamming window is better than that of the rectangular window. The Hamming window attenuates the speech signal at the edge of the window, effectively solving the problem of rectangular windows losing details. Usually, for frequency domain processing of speech signals, the Hamming window is used as the window function. Therefore, when analyzing speech signals, this paper chooses the Hamming window, which can be obtained by stacking the spectra of three rectangular windows, commonly known as the raised cosine window. Its main lobe width reduces frequency resolution. The expression for The Hanning Window (2024) is as Equation (11):


(11) $$w\left(n\right)=\{\begin{array}{c} 0.5\left[1-\cos\left(\frac{2\pi }{N-1}\right)\right],\;0\leq n\leq N-1\\ 0,\;\mathrm{other} \end{array}$$

After windowing, the voice signal forms many frames.

#### 2.2.3 Speech signal endpoint detection

Endpoint detection is a fundamental component in speech processing, playing a vital role in both speech recognition and speech comparison tasks. Its importance cannot be overstated, as it serves two primary functions that significantly impact the efficiency and accuracy of the processing workflow. First, endpoint detection is crucial for distinguishing between meaningful speech segments and periods of silence or background noise. By accurately identifying the start and end points of speech within a signal, endpoint detection helps filter out irrelevant data. This process involves removing non-speech elements such as noise and pauses, which often clutter the signal and complicate subsequent analysis (Ahmed and Lawaye, 2023). This filtering is essential for focusing on the actual spoken content, thereby enhancing the quality of the data being analyzed and improving the reliability of the results. Second, effective endpoint detection accelerates the feature extraction process. By clearly defining the boundaries of speech segments, it allows for the extraction of features from only the relevant portions of the signal. This targeted approach not only streamlines the feature extraction process but also reduces computational load and processing time (Liang et al., 2023). As a result, the overall efficiency of the speech processing system is improved, enabling faster and more accurate analysis. In this paper, an endpoint detection algorithm with enhanced noise resistance is utilized. This algorithm integrates two key techniques: short-term energy analysis and spectral subtraction. Short-term energy analysis evaluates the energy levels of the speech signal over short intervals, helping to identify active speech regions. Spectral subtraction, on the other hand, helps to reduce the influence of noise by subtracting an estimate of the noise spectrum from the speech spectrum. The combination of these techniques enhances the algorithm’s ability to accurately detect speech endpoints even in noisy contexts, leading to more precise and effective speech processing outcomes.

Divide the speech signal into frames, perform square operations on each frame’s signal, and sum up to obtain short-term energy. The formula is as Equation (12) (Huang et al., 2001):


(12) $$E\left(n\right)=\sum {\left(x_{i}\right)}^{2}$$

Among them, $x_{i}$ represents the i -th sample point.

Set an energy threshold based on the actual situation to determine whether the current frame is a speech frame. In a short-term energy sequence, find the first point that continuously exceeds the energy threshold as the starting point. In a short-term energy sequence, starting from the starting point and looking back, find the first point that is continuously below the energy threshold as the ending point. To ensure more accurate endpoint position, trace forward or move back a certain number of frames.

Perform Fast Fourier Transform (FFT) on each frame and calculate the amplitude spectrum using Equation (13) (Shen and Wai, 2022):


(13) $$M\left(n,k\right)=|X\left(n,k\right)|$$

Among them, $X\left(n,k\right)$ represents the complex representation of the k -th frequency point in frame n .

Select frames from non-speech segments, calculate the average value of their spectrum as the noise spectrum, and the formula is as Equation (14) (Shen and Wai, 2022):


(14) $$N\left(k\right)=\frac{1}{M\times \sum M\left(n,k\right)}$$

Perform spectral subtraction on each frame, subtract the amplitude spectrum of the current frame from the noise spectrum, and take the absolute value of the result to obtain the amplitude spectrum after spectral subtraction. Determine whether the current frame is a speech frame by comparing the average energy of the amplitude spectrum values processed by spectral subtraction with the threshold. In the frames identified as speech frames, find the first and last frames as the starting and ending points, adjust the endpoint position to improve the accuracy of the endpoints.

From the above analysis, it can be seen that pre emphasis can weaken the attenuation of high-frequency signals during transmission, improve the energy of the high-frequency part of speech signals, and help improve the clarity and accuracy of sound; Normalization can eliminate audio amplitude differences between different sentences or utterances, making each segment of audio have the same energy level, making it easier for feature extraction and model training; Framing and windowing can segment continuous speech signals into short frames, while windowing helps eliminate boundary effects and facilitates subsequent feature extraction and analysis; Endpoint detection can accurately determine the starting and ending points of speech signals, avoiding the interference of noise and silence on speech recognition and analysis, thereby improving teaching effectiveness and learning experience. The integration of these processing steps makes English pronunciation speech signals more standardized and stable, providing a better foundation for speech features and signal quality assurance for ESL teaching.

### 2.3 Feature extraction of speech signals

Building upon the preprocessing of English pronunciation speech signals, this approach involves extracting and analyzing key features—accuracy, fluency, and completeness—to evaluate students’ pronunciation quality. Accuracy assesses how closely students’ pronunciation aligns with target phonetic standards, focusing on the correctness of individual sounds and phoneme production. Voice fluency verification reflecting how effortlessly students speak without undue hesitation. Completeness evaluates whether students effectively convey the full intended meaning of their speech, including correct stress, intonation, and rhythm. These features are then fused to provide a comprehensive evaluation of pronunciation performance, offering a nuanced view that goes beyond isolated feature assessments. This integrated evaluation serves as a valuable reference for educators, enabling them to refine instructional strategies, implement targeted practice exercises, and enhance the overall effectiveness of English pronunciation teaching. To comprehensively measure the quality of students’ pronunciation, rating features are extracted from three aspects: (1) accuracy features (logarithmic posterior probability, GOP); (2) Fluency characteristics (speech speed, segment duration, and pause duration); (3) Integrity feature (word matching degree).

#### 2.3.1 Accuracy characteristics

Firstly, starting from the levels of logarithmic posterior probability and GOP, the accuracy features of student pronunciation are extracted.

For phoneme $q_{i}$ , its corresponding observation vector for each frame is $O_{t}$ , and the frame level posterior probability is defined as Equation (15) (Rabiner and Juang, 1993; Jelinek, 1998):


(15) $$P\left(q_{i}|O_{t}\right)=\frac{P\left(O_{t}|q_{i}\right)P\left(q_{i}\right)}{\sum_{i=1}^{M}P\left(O_{t}|q_{i}\right)P\left(q_{i}\right)}$$

Among them, $P\left(q_{i}\right)$ represents the prior probability of $q_{i}$ ; $P\left(O_{t}|q_{i}\right)$ represents the likelihood of the observation vector $O_{t}$ given $q_{i}$ ; M represents the total number of factors within the acoustic space.

Assuming $\tau_{i}$ is the starting time of $q_{i}$ and $d_{i}$ is the duration of $q_{i}$ , then the logarithmic posterior probability score ϖ of factor $q_{i}$ is the mean logarithmic posterior probability score of all frame series in $q_{i}$ which is presented in Equation (16) (Rabiner and Juang, 1993):


(16) $${\bar{\varpi }}_{i}=\frac{1}{d_{i}}\overset{\tau_{i}+d_{i}-1}{\underset{t=\tau_{i}}{\sum }}\mathrm{logP}\left(q_{i}|O_{t}\right)$$

The logarithmic posterior probability score ϖ is defined as the mean logarithmic posterior probability score of all N phonemes in the sentence based on Equation (17) (Rabiner and Juang, 1993):


(17) $$\varpi =\frac{1}{N}\overset{N}{\underset{i=1}{\sum }}{\bar{\varpi }}_{i}$$

GOP is a simplification of logarithmic posterior probability, and the GOP of phoneme $q_{i}$ is defined as Equation (18) (Rabiner and Juang, 1993):


(18) $$G_{i}=\log\frac{P\left(q_{i}|O_{t}\right)}{\max_{i\in M}^{P\left(q_{i}|O_{t}\right)}}$$

The GOP score of a sentence can be obtained by taking the average GOP score of all N phonemes as Equation (19) (Rabiner and Juang, 1993):


(19) $$G=\frac{1}{N}\overset{N}{\underset{i=1}{\sum }}G_{i}$$

#### 2.3.2 Fluency characteristics

Speech speed is defined as the number of phonemes a student reads per unit of time, and the speed of speech reflects the fluency of the student’s reading. The calculation formula for speech speed $R_{S}$ is presented in Equation (20) (Zhu et al., 2024):


(20) $$R_{S}=Q/T$$

Among them, T represents the reading time; Q represents the number of phonemes read by students during this period.

The duration of a segment represents the duration of pronunciation for different phonemes in a student’s pronunciation, and the segment duration rating is as Equation (21) (Rabiner and Juang, 1993):


(21) $$D=\frac{1}{N}\overset{N}{\underset{i=1}{\sum }}\log P\left(f\left(d_{i}\right)|q_{i}\right)$$

Among them, $f\left(d_{i}\right)$ represents the normalization function.

When students read aloud, if they are not clear about how to pronounce a word, there will be pauses between the words, and the proportion of the total pause time in the reading time reflects the fluency of the student’s reading. The duration of pause is as Equation (22) (Yuen et al., 2023):


(22) $$D_{PAU}=T_{SIL}/T$$

Among them, $T_{SIL}$ represents the total duration of the silent part in the spoken speech.

#### 2.3.3 Integrity features

When students read aloud, there is a possibility of missing words. The proportion of the number of words read by students in the reading content is used as the evaluation index for completeness, and word matching is defined as Equation (23) (Yuen et al., 2023):


(23) $$R_{\mathrm{MAT}}=\omega /W$$

Among them, ω represents the number of words that match the recognition result with the specified reading content; W represents the total number of words read aloud.

### 2.4 Multi feature fusion of speech signals

A feedback neural network that can fuse multi granularity features is proposed for the accuracy, fluency, and completeness features extracted above, for the fusion of multiple features in speech signals. Feature fusion plays a crucial role in the research of innovative methods for English pronunciation teaching in ESL contexts. By fusing features of different granularities and types such as accuracy, fluency, and completeness, the characteristics of speech signals can be more comprehensively described, thereby improving the accuracy and sensitivity of evaluation platforms to student pronunciation performance. At the same time, multi feature fusion also helps to comprehensively consider the correctness, fluency, and coherence of pronunciation, making the platform more comprehensive in evaluating students’ pronunciation level, providing teachers with more targeted feedback and guidance, and improving students’ learning effectiveness and teaching quality.

**Figure 1:**
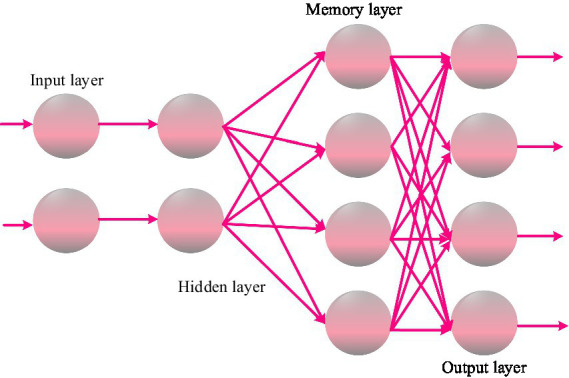
RNN network structure diagram.

RNN adds a feedback mechanism on the basis of artificial neural networks. RNN networks include input layers, hidden layers, memory layers, and output layers. The input layer inputs accuracy features, fluency features, and completeness features, respectively. The memory layer is a collection of neurons fed back from the hidden layer, used to record the content of the previous moment in the hidden layer (He, 2021; Gui, 2024). The structure of the RNN network is shown in Figure 1.

Let t be the current moment where the network is located, $f\left(t\right)$ represent the current speech frame features, $s\left(t\right)$ represent the segment features within the t time period, $E\left(t\right)$ represent the cognitive window features, and $x\left(t\right)$ and $z\left(t\right)$ represent the outputs of the two hidden layers of the network, respectively. $W_{1}$ is the weight matrix connecting input layers $f\left(t\right)$ and $s\left(t\right)$ to hidden layer $x\left(t\right)$ , $W_{2}$ is the weight matrix connecting hidden layer $x\left(t\right)$ to hidden layer $z\left(t\right)$ , $W_{3}$ is the weight matrix connecting hidden layer $z\left(t\right)$ to output layer $y\left(t\right)$ , $W_{4}$ is the weight matrix connecting memory layer $x_{c}\left(t\right)$ to hidden layer $x\left(t\right)$ , and $W_{5}$ is the weight matrix connecting cognitive window feature input layer $E\left(t\right)$ to hidden layer $z\left(t\right)$ . The output of the hidden layer $x\left(t\right)$ is shown in Equation (24):


(24) $$x\left(t\right)=f\left(W_{1}s\left(t\right)+f\left(t\right)\right)+W_{4}x_{c}\left(t\right)$$

Among them, $f\left(\cdot \right)$ takes the sigmoid function. The calculation formula for memory layer $x_{c}\left(t\right)$ is as Equation (25):


(25) $$x_{c}\left(t\right)=x\left(t-1\right)$$

The calculation formula for the hidden layer $z\left(t\right)$ is as Equation (26):


(26) $$z\left(t\right)=f\left(W_{2}x\left(t\right)\right)+W_{5}E\left(t\right)$$

The calculation formula for the hidden layer $y\left(t\right)$ is as Equation (27):


(27) $$y\left(t\right)=f\left(W_{3}z\left(t\right)\right)$$

**Figure 2:**
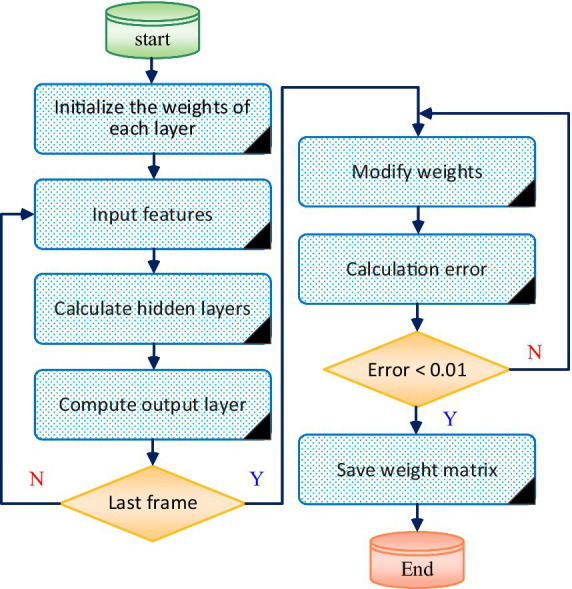
RNN training process.

The classic Back Propagation Error (BP) algorithm (Mirsadeghi et al., 2021; Pommé and Pelczar, 2021; Fatema et al., 2022) is used to update the weights of each layer node in the RNN network. Using the updated weights to expand RNN network training, the training process is shown in Figure 2.

The process of using a trained RNN to achieve multi feature fusion of speech signals can be described as follows: Firstly, different types of features such as accuracy, fluency, and completeness are input into the pre trained RNN network. Through the multi-layer neural structure of the network, learning and extraction of these features are achieved. RNN networks can effectively capture temporal and contextual information between features, thereby better expressing the features of speech signals. Next, through the output layer or related connection layer of the network, different levels of features are fused, and the automatic learning and weight adjustment mechanism of the neural network is utilized to achieve feature synthesis and fusion. Finally, through the backpropagation and optimization process on the training dataset, the network parameters are continuously optimized, enabling the network to accurately fuse different features and extract more representative speech features, providing an effective method for multi feature fusion of speech signals.

### 2.5 Computer assisted English pronunciation evaluation

**Figure 3:**
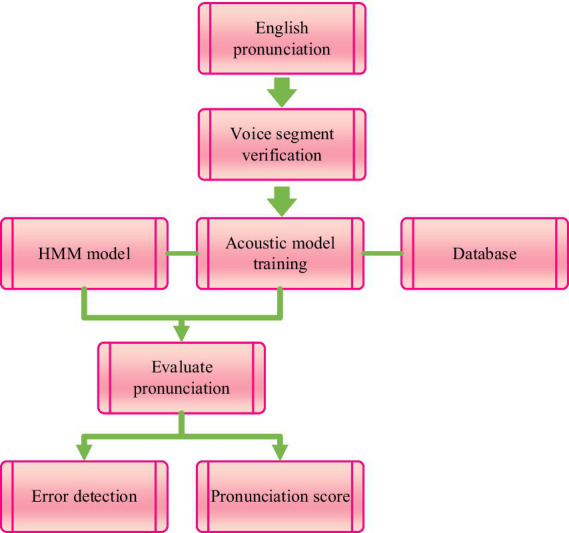
English pronunciation evaluation process.

Based on the multi feature fusion results of English speech signals, computer-aided English pronunciation evaluation is carried out to provide reference for English pronunciation teaching. The English pronunciation evaluation process is shown in Figure 3.

From Figure 3, it can be seen that first, the preprocessed learner’s English pronunciation is subjected to speech segment validation, including vowel segment segmentation, establishment of validation system, and reliability of validation system; Then, the HMM model is used to train acoustic models on a large number of standard pronunciation databases. The Viterbi algorithm is used to segment and decode speech, and this information, including the extraction of evaluation parameters, regularization of evaluation parameters, parameter association process, and evaluation mechanism, is sent to the core of the English pronunciation evaluation system, which is the pronunciation evaluation module. Through this module, the weights of each evaluation parameter in English pronunciation evaluation are calculated, to reflect the opinions of human experts on the quality of English sentences and provide feedback to learners, including comprehensive ratings and corrective suggestions compared through expert knowledge bases.

## 3 Experimental verification analysis

### 3.1 Experimental data

Use the Sphinx4 speech recognition system released by Carnegie Mellon University as the experimental platform. In order to extract feature parameters that represent the essence of English pronunciation, pre-emphasis, normalization, framing, windowing, and endpoint detection are performed on English speech data, laying the foundation for subsequent feature extraction and pronunciation evaluation. The sample size of this study is the English pronunciation of 100 college students, half male and half female, covering students with different pronunciation levels. They read sentences from the Arctic corpus, each containing 8 to 20 words, totaling 1,000 sets of pronunciation data. The type of English studied is General English.

### 3.2 Evaluation indicators

- Teaching evaluation accuracy: Teaching evaluation accuracy refers to the degree to which a method’s evaluation of student learning outcomes or teaching effectiveness is in line with the actual situation.
- Speech fluency: Set speech fluency to 5 levels, with higher levels indicating higher speech fluency. According to the established fluency assessment criteria, professional speech evaluators or teachers are used as evaluators to rate or grade the spoken language of learners. The evaluation criteria include pronunciation accuracy, speaking speed, intonation, and appropriateness of pauses.Spectral features: Spectral features reveal the characteristics of speech signals in the frequency domain, which are used to verify that the method can effectively extract the essential feature parameters of speech.

### 3.3 Result analysis

#### 3.3.1 Verification of teaching evaluation accuracy

In order to verify the application effect of the method proposed in this paper, the accuracy of teaching evaluation was taken as the experimental indicator. The teaching evaluation accuracy of the method proposed in this paper, the method Wen (2020), and Tuba et al. (2018) were compared, and the results are shown in Table 1.

**Table 1:** Teaching evaluation accuracy test results.

	Accuracy %		
Test no	Wen, 2020	Tuba et al., 2018	Proposed method
1	68.5	72.3	98.0
2	62.9	76.4	96.1
3	65.4	67.9	99.3
4	66.0	64.2	98.6
5	68.9	69.0	97.2
6	73.1	77.8	99.0
7	67.0	70.8	97.8

According to the analysis of Table 1, the highest accuracy value of the teaching evaluation method in this paper can reach 99.3%, the highest accuracy value of the teaching evaluation method Wen (2020) is 68.9%, and the highest accuracy value of the teaching evaluation method Tuba et al. (2018) is 77.8%. And in multiple tests, the accuracy of the method proposed in this paper is higher than that of the methods Wen (2020) and Tuba et al. (2018), indicating that the method proposed in this paper has high evaluation accuracy and can help teachers understand students’ learning situations and optimize teaching strategies.

#### 3.3.2 Voice fluency verification

On the basis of the above results, speech fluency was used as an evaluation indicator to compare and verify the methods Wen (2020) and Tuba et al. (2018), and our method. The results are shown in Table 2.

**Table 2:** Results of speech fluency test.

Test no	Voice fluency		
	Wen, 2020	Tuba et al., 2018	Proposed method
1	4	2	5
2	3	5	4
3	3	4	4
4	4	3	5
5	3	3	5
6	2	4	5
7	5	5	5

According to the analysis of Table 2, it can be seen that under the application of the method in this paper, students have a higher level of fluency in English pronunciation, both reaching level 4 or above. However, under the application of the methods Wen (2020) and Tuba et al. (2018), students have a lower level of fluency in English pronunciation. The application effect of the English pronunciation teaching method proposed in this paper is superior to existing methods, and it helps to improve the fluency of students’ English pronunciation. Therefore, it has higher application value.

#### 3.3.3 Spectral feature verification

**Figure 4:**
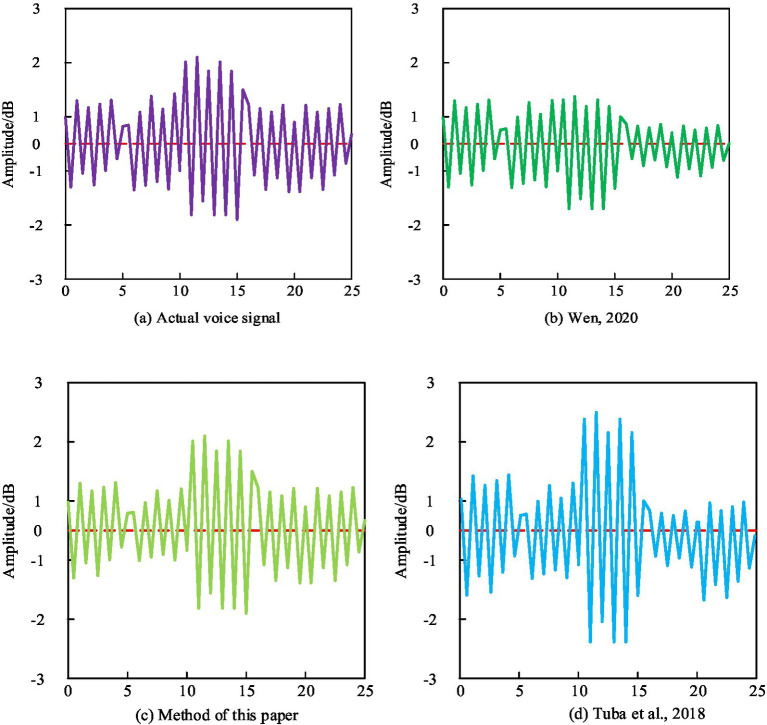
Spectral feature test results.

In English pronunciation teaching, amplitude is used to represent the characteristics of speech signals in the frequency domain. By analyzing the spectral features, we can better understand the energy distribution of speech signals in the frequency domain, which is helpful for pronunciation teaching and speech recognition research. The test results of different methods are shown in Figure 4.

From Figure 4, it can be seen that the amplitude of the English speech signal obtained by the method proposed in this paper has a high similarity with the amplitude of the original speech signal, while the amplitude of the English speech signal obtained by the methods proposed Wen (2020) and Tuba et al. (2018) has a certain difference from the amplitude of the original speech signal. This indicates that the method proposed in this paper can accurately extract the characteristics of student speech signals, which helps teachers to obtain information about their learning status, This is because the method proposed in this paper not only extracts features such as accuracy, fluency, and completeness of student English pronunciation, but also integrates these features to achieve effective evaluation of student pronunciation quality, providing reliable reference for improving the effectiveness of English pronunciation teaching.

## 4 Conclusion

This study presents an innovative approach to teaching English pronunciation in an ESL context, incorporating modern technology and advanced algorithms to enhance the learning experience. The methodology includes multi-sensor detection methods for accurate data collection, preprocessing techniques such as pre-emphasis, normalization, framing, windowing, and endpoint detection to optimize speech signal quality, and the extraction and fusion of features related to pronunciation accuracy, fluency, and completeness using a feedback neural network. Empirical validation of this method shows significant improvements over existing techniques. The teaching evaluation accuracy of the proposed method reached up to 99.3%, compared to 68.9 and 77.8% for methods referenced in previous studies. Furthermore, students demonstrated higher fluency in English pronunciation, with most achieving a fluency level of 4 or above, whereas existing methods resulted in lower fluency levels. The proposed method also excelled in spectral feature verification, accurately extracting speech signal characteristics and aligning closely with the original speech signal amplitudes, unlike the notable discrepancies observed in methods from previous studies. Overall, this innovative approach provides a robust framework for improving English pronunciation teaching effectiveness, offering personalized guidance and real-time feedback, which are critical for addressing individual learner needs and enhancing overall teaching quality.

Future research can further explore the optimization of English oral pronunciation and speech signal detection models, as well as more diverse feature extraction methods, to more comprehensively evaluate students’ English pronunciation ability. At the same time, it is possible to consider integrating artificial intelligence and machine learning technologies more deeply into English pronunciation teaching, achieving more personalized and intelligent teaching guidance. In addition, research can also be conducted on how to combine pronunciation teaching with the teaching of other language skills such as listening, reading, and writing to promote the improvement of students’ comprehensive English abilities.

## Data Availability

The original contributions presented in the study are included in the article/supplementary material, further inquiries can be directed to the corresponding author.

## Glossary

ASR: Automatic speech recognition
BP: Back Propagation
CNN: Convolutional Neural Network
ESL: English as a Second Language
FEA: Finite Element Analysis
FFT: Fast Fourier Transform
GOP: Goodness of Pronunciation
HMM: Hidden Markov Models
IT: Information Technology
LSTM: Long Short-Term Memory
MOLP: Multi-Objective Linear Programming
RNN: Recurrent Neural Network
a: The advanced statistical features of the signal
b: The frequency shift distribution
l: The frame length
M: The total number of factors within the acoustic space
n: The number of voice point
N: The phonemes in the sentence
Q: The number of phonemes read by students
s: The frame shift
T: The reading time
w: The total number of words read aloud
$d\left(t\right)$: The amplitude of the received signal
$f\left(0\right)$: The sigmoid function
$f\left(n\right)$: The frame rate
$f\left(t\right)$: The step transfer function of speech signals
$M\left(n,k\right)$: The amplitude spectrum
$P\left(q_{i}\right)$: The prior probability of $q_{i}$
$P\left(O_{t}|q_{i}\right)$: The likelihood of the observation vector
$s\left(\phi ,\varepsilon \right)$: The output extension of English pronunciation signals
$s\left(t\right)$: The segment features in the t time period
$u\left(t\right)$: The resonance peak
$X\left(n\right)$: The speech signal
$X\left(n,k\right)$: The complex representation of the k -th frequency point in frame n
$x\left(t\right)$: The output of the hidden layer of the network
$x\left[n\right]$: The speech signal
$y\left(t\right)$: The output layer
$y\left(t-\phi \right)$: The complex envelope of English pronunciation frequency components
$z\left(t\right)$: The output of the hidden layer of the network
$d_{i}$: The normalization function
$R_{k}\left(t\right)$: The phase of speech detection
$T_{SIL}$: The total duration of speech silence
$W_{i}$: The weight matrix
$x_{c}\left(t\right)$: The connecting memory layer
$x_{i}$: The i-th sample point
$Y_{k}^{\prime }\left(t\right)$: The output signal component
$z_{n}$: The distribution of sample speech elements
α: The pre-emphasis coefficient
ε: The bandwidth of signal acquisition feature expansion
∅: The frequency shift in English pronunciation signals
ϖ: The mean logarithmic posterior probability score
$\tau_{i}$: The starting time
$\mu \left(a,b\right)$: The delay component of broadband signal
